# Design and psychometric evaluation of RES-PRIM: a resilience scale for primary education students with and without neurodevelopmental disorders

**DOI:** 10.3389/fpsyt.2025.1663460

**Published:** 2025-10-08

**Authors:** Raquel Flores-Buils, Clara Andrés-Roqueta, Rosa Mateu-Pérez

**Affiliations:** ^1^ Department of Developmental, Educational Social and Methodological Psychology, Universitat Jaume I, Castellón de la Plana, Spain; ^2^ Department of Pedagogy and Didactics of Social Sciences, Language and Literature, Universitat Jaume I, Castellón de la Plana, Spain

**Keywords:** resilience, children, primary education, typical development, neurodevelopmental disorders, individual and contextual protective factors, quality of life, psychometric evaluation

## Abstract

**Background:**

Promoting resilience is a proven pathway to well-being, participation, and quality of life in childhood; it is particularly critical for learners with neurodevelopmental disorders (NDD), who encounter layered academic and socio-emotional challenges. Yet existing resilience measures rarely target the 6- to 12-year age band and none offer the inclusive, visually supported format required by many neurodivergent pupils.

**Objective:**

To design and provide evidence of validity and reliability for RES-PRIM, a child-friendly, picture-augmented scale that captures both individual strengths (e.g., self-esteem, problem-solving) and contextual supports (e.g., family, peer, and teacher backing) in children with and without NDD.

**Method:**

After an evidence-guided item-generation process rooted in universal-design principles, RES-PRIM was administered to 529 Spanish primary-school students (465 typically developing, 64 with NDD). Confirmatory factor analysis (CFA) was conducted to provide evidence of validity regarding internal structure, and reliability was examined for the overall scale and each factor using Cronbach’s alpha and McDonald’s omega. In addition, external measures of emotional regulation and academic stress were applied to analyze evidence of relations to external variables.

**Results:**

CFA supported a nine–first-order/two–second-order structure with excellent fit (χ²/df = 1.61, RMSEA= .038, SRMR= .045, CFI= .934, TLI= .922). Reliability was satisfactory for the total scale and all dimensions, with Cronbach’s alpha ranging from.70 to.87 and McDonald’s omega from.72 to.88. Evidence of relations to external variables emerged through the expected associations: higher resilience correlated with better emotion regulation and lower academic stress.

**Conclusions:**

RES-PRIM provides researchers and practitioners with a robust, inclusive assessment tool that can (a) identify resilience profiles in diverse classrooms, and (b) guide evidence-based, multi-tiered interventions aimed at enhancing children’s quality of life and full participation.

## 1 Introduction

Quality of life (QoL) in children is influenced by their physical health, emotional well-being, the quality of their social relationships, and their level of participation in meaningful and inclusive activities (1). For decades, the scientific literature has focused on the consequences of exposure to adverse childhood events—such as maltreatment, school difficulties, the loss of a loved one, parental mental illness, or family separation (2, 3). These experiences increase the likelihood of developing mental-health problems such as anxiety, depression, or post-traumatic stress disorder (4).

Although resilience has been recognized as a key protective factor against these risks, there is still a lack of appropriate assessment tools for primary-school children, particularly those with neurodevelopmental disorders (NDD). Most available resilience measures were developed for adolescents or adults, and do not address the specific needs of younger children or those requiring additional educational support (5–7). This gap highlights the importance of designing accessible, developmentally appropriate, and multimodal instruments for this population.

Having appropriate resilience assessment tools is especially relevant for children with neurodevelopmental disorders (NDD), since in addition to adverse experiences they also face specific developmental difficulties. The term NDD encompasses chronic clinical conditions of early onset, such as Communication Disorders, Attention-Deficit/Hyperactivity Disorder (ADHD), and Autism Spectrum Disorder (ASD) (8). In the present study, the validation of RES-PRIM focused on primary-school children both with typical development and with NDD, specifically ADHD and ASD, given their prevalence in school-age populations and their frequent presence in inclusive classroom settings. While the instrument was designed to be accessible to primary-school children in general, the current evidence of validity is limited to these two diagnostic groups within the NDD spectrum. These disorders are characterized by atypical development in various socio-emotional, cognitive, motor, and/or language skills, often leading to difficulties in the academic sphere (e.g., reading, writing, or math problems) and in personal domains (e.g., social-relationship difficulties, emotional understanding, or risk of abuse). Moreover, children with NDD have a greater predisposition to developing secondary psychopathological disorders such as anxiety, depression, or self-regulation problems (9). This can generate frustration, feelings of difference, and/or peer rejection, negatively affecting their self-esteem and confidence as they grow and become aware of their challenges (10).

Research has shown that, in both typically developing children and those with NDD, resilience acts as a key protective factor that boosts quality of life and subjective well-being. By strengthening emotional self-regulation, creative problem solving, and the seeking of social support, resilience benefits multiple domains: it lowers perceived stress, increases the sense of belonging, and facilitates active participation in school and community settings, leading to better academic and behavioral outcomes (9, 11–16). Resilience is generally defined as the capacity of an individual to adapt positively to adverse events and to project themselves into the future (17, 18). More recently, the American Psychological Association (APA) (19) emphasized its role in enabling emotional regulation and effective stress management, improving children’s adaptability in the face of challenges.

However, despite the relevance of this construct, prior reviews have consistently highlighted conceptual and methodological challenges in defining and assessing resilience, including a lack of agreement on its operationalization and limited availability of technically adequate measures (5, 7, 20). In particular, most resilience instruments have been developed for adolescents or adults, with scarce validated tools tailored for primary-school children.

Resilience is not a fixed trait but a dynamic process involving the interaction of individual and contextual factors (10). Among the individual factors, self-esteem, self-perception, emotional well-being, optimism, social skills, and self-regulation stand out. Recent studies examining resilience in children with ADHD and ASD have found that are able to develop protective factors that allow them to cope positively with adversity (9, 10). Although ADHD has traditionally been studied from a deficit perspective, recent research has identified that some children with ADHD may possess advanced verbal skills, well-developed logical thinking, and effective coping strategies (15).

Among contextual factors, social support is crucial for resilience development. Support from friends, family, and the school community has been shown to be fundamental in improving coping skills and reducing vulnerability to stress, anxiety, and depression in children with and without NDD (21). However, children with ASD and ADHD are more exposed to adverse experiences, such as social rejection and academic difficulties, which can hinder resilience development (9). Studies have demonstrated that resilience in these children can improve academic performance, facilitate social inclusion, and strengthen mental well-being (15, 22).

Because resilience can be developed over time, it is crucial to implement strategies that strengthen it from childhood, as these reduce the impact of adverse situations and contribute to preventing mental-health disorders. To design such strategies effectively, children’s resilience must first be measured in relation to the individual and contextual factors that contribute to it. Having valid and developmentally appropriate assessment instruments is therefore essential for promoting resilience and improving adaptation and quality of life from an early age (3, 9, 17).

Although broader multi-informant systems, such as the Social and Emotional Assets and Resilience Scales (SEARS), include parent-, teacher-, and self-report formats to assess socio-emotional competencies including resilience (23), there are currently no instruments specifically designed to measure resilience in primary school children. This gap underlines the importance of developing self-report measures adapted to this age group, as they allow children to express their own perceptions of strengths and supports, which are central to resilience and subjective well-being.

Moreover, most existing resilience measures have been developed and validated in English-speaking contexts (5, 7), raising concerns about their cultural and linguistic applicability to other populations. In this regard, the present work was developed in Spain and specifically designed in Spanish, to ensure contextual and linguistic appropriateness for primary-school children. According to the International Test Commission (ITC) Guidelines for Translating and Adapting Tests (2017), direct translation without cultural adaptation may compromise validity, which further highlights the importance of creating resilience measures adapted to the Spanish context and language, particularly for children with NDD.

Children in primary education, and particularly those with specific educational support needs, benefit from visual supports that complement textual information. Research shows that such supports enhance task comprehension, sustain attention, and increase motivation during assessment tasks (24, 25). Moreover, when combined with structured procedures, visual schedules have demonstrated efficacy in improving task performance across diverse settings (26). Visual prompts have also been shown to reduce noncompliance during transitions (27). Therefore, the inclusion of images in assessment instruments is a valuable strategy to enhance accessibility and response accuracy across all primary-school children, not only those with NDD.

Currently, the instruments available to measure resilience in children are limited, especially for those with NDD. Most existing scales are designed for children older than nine, such as the Healthy Kids Resilience Assessment (28), the Personal Resilience Factors Inventory (29), and the Child and Youth Resilience Measure (CYRM-28, 30). Although the latter has recently been adapted for children as young as five (CYRM-12, 21), these instruments have provided evidence of validity primarily in typically developing or community samples, not specifically in populations with NDD. Moreover, they rely exclusively on textual items, which may hinder comprehension in younger children or those with language difficulties. At present, there are no multimodal assessment tools adapted to the characteristics of children with NDD that incorporate visual supports, such as images, which, as noted above, are highly useful for sustaining attention, enhancing motivation, and facilitating item comprehension.

Given the lack of suitable instruments, together with the absence of culturally and linguistically adapted measures for Spanish primary-school children, and the need to effectively assess resilience young children with and without neurodevelopmental disorders (NDD), the present study aims to design and provide validity and reliability evidence for the RES-PRIM, a scale for evaluating individual and contextual resilience factors in primary-school children, both with typical development and with NDD.

## 2 Method

### 2.1 Design

This study followed an instrumental design, as defined by Montero and León (31). The research process was structured into four sequential stages: (a) procedure, describing the steps followed for the construction, piloting, and implementation of the instrument, aimed at providing evidence of validity based on internal structure and on relationships with other variables; (b) participants, detailing the study sample; (c) instrument development, including the RES-PRIM and the additional measures administered (the IECI and TEC, selected to test relations to external variables); and (d) data analysis, specifying the statistical techniques applied.

### 2.2 Procedure

**Figure 1:**
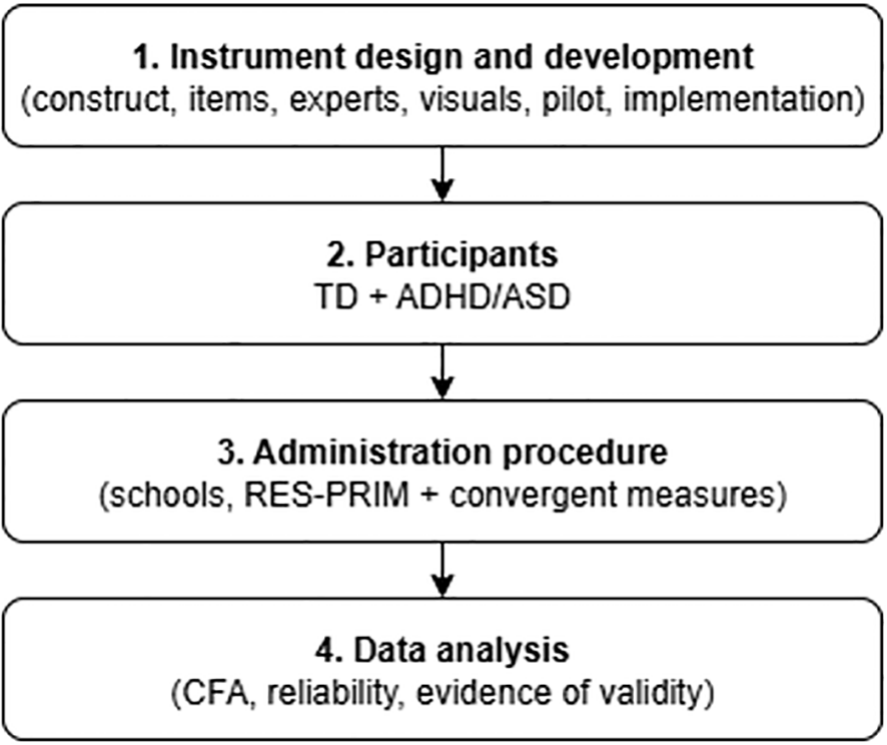
Methodological sequence of the study.

Following the recommendations for instrumental studies (31) and international guidelines for test development (32), the construction and validation of the RES-PRIM proceeded through sequential stages, each aimed at providing specific sources of validity evidence. A schematic summary of these stages is presented in **Figure 1**.

#### 2.2.1 Construct definition and dimension specification

For the design of the RES-PRIM, a comprehensive review of the scientific literature on resilience in childhood was conducted. This review included theoretical models that explain the resilience process and its promotion in educational contexts. Among them, Bronfenbrenner’s Ecological Model (33) highlights the multi-level environmental influences on child development; Masten and Obradovic’s Resilience Model (34) conceptualizes resilience as a dynamic process of adaptation to adversity; and the Integrative Resilience Model by Mateu, Gil, and García-Renedo (35) offers a multidimensional view combining individual and contextual factors. Likewise, applied models such as Grotberg’s Resilience Promotion Guide (36), Vanistendael’s Resilience Building Model (37), and Henderson and Milstein’s Resilience Wheel (38) were reviewed, as they provide concrete strategies for fostering resilience in school settings.

Drawing on these theoretical and applied models, as well as on empirical studies on factors influencing the development of resilience, two broad domains were specified with the following specific factors:

- Individual factors, including empathy/prosocial behavior (39, 40), social skills (41), self-esteem (42), emotional introspection (43), problem-solving skills (14), and future orientation/purpose in life (44, 45).
- Contextual factors, referring to protective supports in the child’s environment, namely adult support at home (46), adult support at school (47), and peer support (48).

This blueprint ensured that the scale covered the most relevant dimensions of resilience identified in the literature, integrating personal strengths and environmental supports. By combining these perspectives, the RES-PRIM was designed to capture resilience as a dynamic and multidimensional process, consistent with contemporary theoretical and applied approaches.

#### 2.2.2 Item writing and format

Items were initially generated by a first group of three experts in resilience in educational contexts, who drafted a preliminary pool of 40 items. Statements were written in clear, age-appropriate language, ensuring that primary-school children could easily read and understand them without requiring adult mediation. Each item referred to everyday situations and was framed in the first person. To facilitate accessible responses, a 4-point Likert scale was used (*3 = always, 2 = many times, 1 = few times, 0 = never*). Special care was taken to avoid double negatives, jargon, or multi-barrel constructions, and to keep sentences short and concrete.

**Figure 2:**
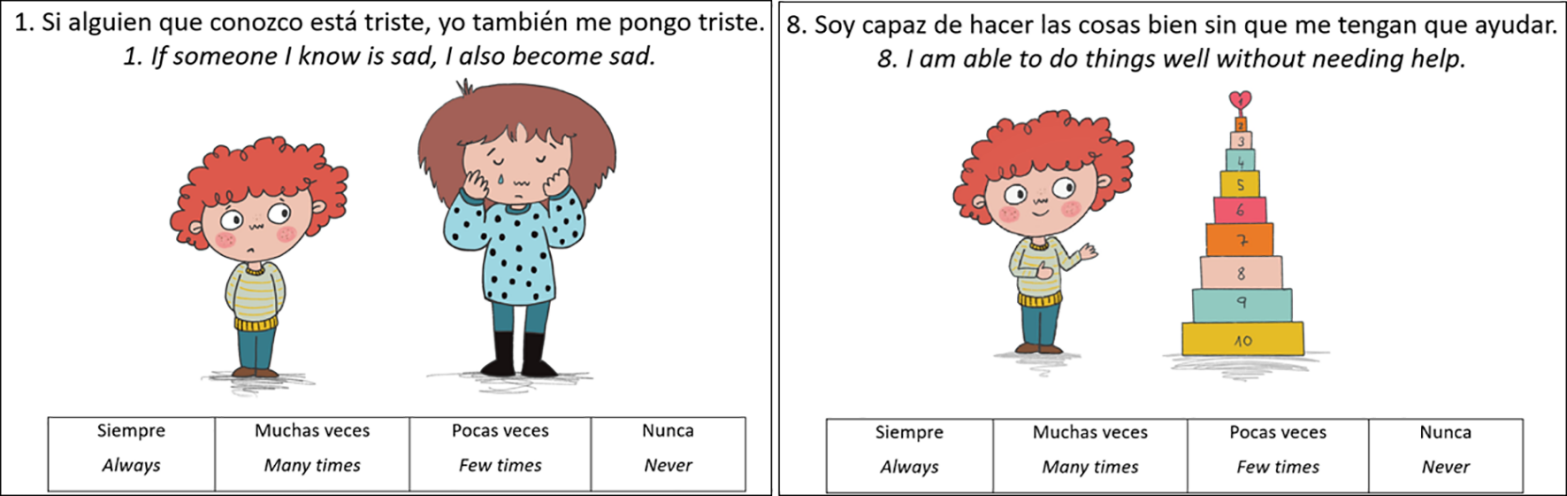
Examples of items on individual factors.

**Figure 3:**
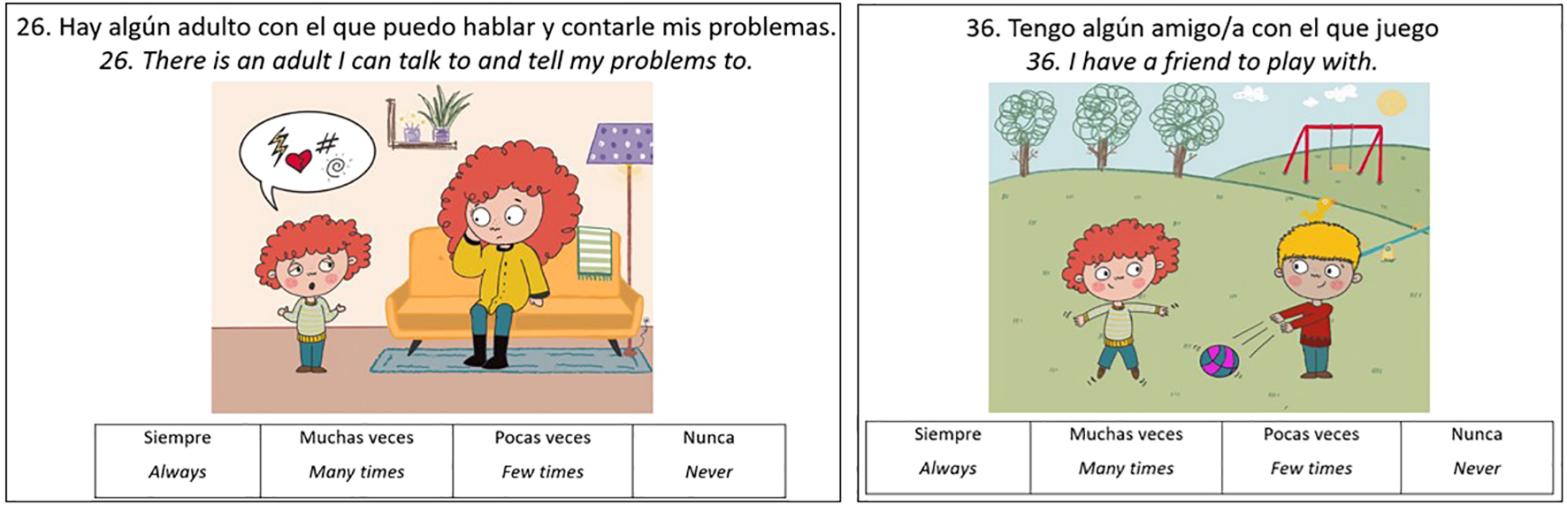
Examples of Items on Contextual Factors.

A distinctive feature of the RES-PRIM is that each item was accompanied by a visual illustration (see **Figures 2**, **3**). These images were designed to clarify the intended meaning, reduce linguistic load, and support comprehension for children with diverse educational needs. Visual supports served multiple functions: Improving comprehension of abstract or complex statements by anchoring them in familiar, concrete situations (49); Facilitating interpretation for children with neurodevelopmental conditions such as ASD or language disorders, who may struggle with abstract verbal information (50); Sustaining attention and engagement in children with ADHD, as images act as visual anchors that help maintain focus during the task; and, Promoting inclusivity, by ensuring that all children, regardless of language proficiency or developmental profile, can access the assessment under equitable conditions.

In line with the theoretical framework established in the construct definition stage, and after the subsequent expert review process, the final pool of 40 items was distributed across two domains:

- Individual resilience factors, assessing empathy/prosocial behavior, social skills, self-esteem, emotional introspection, problem-solving, and future orientation.
- Contextual resilience factors, covering adult support at home, adult support at school, and peer support.

Together, the wording and visual presentation of the items aimed to create a child-friendly and developmentally sensitive assessment tool, consistent with universal design principles.

#### 2.2.3 Expert review (content validity)

The preliminary pool of 40 items with visual illustrations was subsequently submitted to a second panel of experts, composed of two educational psychologists and twelve teachers from preschool and primary education. The purpose of this stage was to gather content validity evidence by ensuring that the items effectively represented resilience-related constructs, while also being accessible and understandable to the target age group. Experts rated each item and its corresponding illustration on a 0–5 scale in terms of clarity (precision of wording), comprehensibility (ease of understanding for children), and appropriateness (relevance for assessing resilience in school contexts). They also provided qualitative comments on potentially problematic items.

Quantitative ratings and qualitative feedback were jointly considered to guide item refinement. Based on the results, four items were reformulated to improve wording and ensure closer alignment with their illustrations, thus enhancing both interpretability and content validity. This stage therefore provided robust evidence of content validity, combining the perspectives of researchers and practitioners to guarantee that the RES-PRIM items were theoretically grounded, developmentally appropriate, and practically useful for diverse school populations.

#### 2.2.4 Pilot testing (comprehension, feasibility, and item refinement)

After incorporating expert feedback, the preliminary 40-item version of the scale was administered to a pilot group of 18 primary-school children, including 12 with typical development and 6 with NDD (3 with ASD and 3 with ADHD). The aim of this stage was to evaluate comprehension, feasibility, and response process in real classroom conditions.

During administration, trained examiners observed children’s reactions and recorded any signs of confusion, hesitation, or requests for clarification. In addition, brief probe questions were used to verify whether children correctly understood the meaning of selected items and their corresponding illustrations.

The pilot testing confirmed the overall adequacy of the scale format and visual supports. Several minor wording adjustments were introduced to simplify specific items, and some illustrations were refined to ensure they matched children’s interpretations more accurately.

This phase therefore provided evidence of face validity and response process validity, and confirmed that the instrument was developmentally appropriate and feasible for administration to the target population. In addition, pilot testing also verified children’s comprehension of the accompanying illustrations, which were refined when necessary to ensure clarity and cultural appropriateness.

#### 2.2.5 Implementation of the scale in the main study

After the pilot stage, the 40-item version of the RES-PRIM was administered to the main study sample in the school setting. Administration procedures varied depending on grade level. For first- and second-grade students, the application was individual, through one-on-one sessions conducted by a trained member of the research team, who read the items aloud to each child to address possible limitations in reading comprehension. For third- to sixth-grade students, the administration was group-based in classrooms, with two members of the research team present to supervise the process and assist children if any clarification was needed.

The scale items were accompanied by illustrations of everyday situations, in order to ensure that the instrument was comprehensible and engaging for all children, regardless of their cognitive or linguistic profile. The approximate duration was 20–30 minutes in the group modality and 30–40 minutes in the individual modality. School personnel collaborated by facilitating parental authorization procedures, organizing the logistics of data collection, and coordinating with the research team; however, the questionnaires were administered exclusively by trained members of the research team.

Finally, the study complied with all required ethical standards. Approval was obtained from the university’s ethics committee and permission was granted by the regional educational administration to conduct the research in schools. Additionally, meetings were held with school management teams to explain the study’s objectives and obtain their collaboration. Informed consent was secured from the parents of all participants, and confidentiality of the collected data was ensured.

As detailed in the Data analysis section, two items were later removed on psychometric grounds after examining the full dataset, resulting in a final operational version with 38 items. The subsequent Confirmatory Factor Analysis (CFA) was conducted to provide evidence of validity regarding the internal structure of the instrument.

### 2.3 Participants

A non-probabilistic convenience sampling method was used for the present study. Participants were recruited through collaboration with school management teams from several public primary schools in the Valencian Community (Spain), which voluntarily agreed to participate after being contacted by the research team. The final sample comprised 529 students (238 boys [51.18%], 227 girls [48.81%]) aged between 5 and 12 years (M=8.89, SD=1.79). Inclusion criteria were: (a) being enrolled in primary education (ages 6–12) and (b) providing written informed consent from parents/guardians together with assent from the child.

Of these, 465 children were typically developing (TD) and 64 presented a neurodevelopmental disorder (NDD). The NDD group included 32 children with a diagnosis of Level 1 Autism Spectrum Disorder (ASD) and 32 with Attention-Deficit/Hyperactivity Disorder (ADHD). All attended mainstream classrooms and had been previously diagnosed by licensed clinical professionals according to DSM-5 criteria, following regional protocols. For ASD, diagnostic reports confirmed Level 1 classification based on standardized instruments such as the Autism Diagnostic Interview-Revised (ADI-R) and the Autism Diagnostic Observation Schedule (ADOS) (51). For ADHD, diagnoses were established using standardized measures commonly employed in Spanish clinical and educational contexts, including the Conners’ Rating Scales (52) and the AULA Nesplora continuous performance test (53, 54). Families provided diagnostic documentation, which was verified by the schools’ educational support services. Although two children had reports noting both ASD and ADHD, no additional comorbidities were recorded.

Given the relatively small size of each subgroup, and following an inclusive approach, students with ASD and ADHD were grouped together under the umbrella category of NDD for the main analyses. However, their separate distributions are also reported in **Table 1** to provide a more detailed description of the sample.

**Table 1:** Description of the participants.

Course	n TD	N NDD	n ASD	n ADHD	n Total	Age	n sex (%)	
						M (SD)	Boys	Girls
1st	69	10	5	5	79	6.17 (.382)	41 (50.7%)	38 (49.3%)
2nd	78	8	4	4	86	7.27 (.446)	48 (55.1%)	38 (44.9%)
3rd	64	13	6	7	77	8.38 (.488)	46 (56.3%)	31 (43.8%)
4th	81	9	5	4	90	9.15 (.357)	39 (40.7%)	51 (59.3%)
5th	80	11	5	6	91	10.23 (.420)	49 (53.8%)	42 (46.3%)
6th	93	13	7	6	106	11.26 (.440)	57 (51.6%)	49 (48.4%)
Total	465	64	32	32	529	8.89 (1.788)	238 (51.18%)	227 (48.81%)

### 2.4 Instrument

#### 2.4.1 Resilience scale for primary school students

Resilience Scale for Primary School Students (RES-PRIM). The RES-PRIM is a novel instrument originally developed in Spanish consisting of 38 self-report items to measure resilience in children. It was explicitly designed as a self-report questionnaire, ensuring that responses reflect the child’s own perspective rather than relying on external informants such as parents or teachers. In accordance with children’s reading skills, the instrument was administered in two formats: younger students (1st–2nd grade) responded individually with the items read aloud by a trained researcher, while older students (3rd–6th grade) completed the questionnaire in classroom groups under researcher supervision. The items were developed *de novo* by a panel of experts in resilience in educational contexts, based on the nine theoretically grounded dimensions (six individual and three contextual factors) identified during the construct definition stage. The present study is part of the initial validation process of the RES-PRIM, and therefore its psychometric properties are examined here.

Each item is a simple declarative statement written in the first person, accompanied by a visual illustration, and rated on a 4-point Likert scale (3 = always, 2 = many times, 1 = few times, 0 = never). For instance, one empathy-related item reads: *“If someone I know is sad, I also become sad,”* accompanied by an illustration of a child looking sad next to a sad peer (see **Figure 2**).

The development process followed the International Test Commission (ITC) Guidelines for Translating and Adapting Tests (55), which emphasize cultural and linguistic adaptation, clarity of item wording, and accessibility for the intended population. Twenty-four items measure individual resilient factors (empathy/prosocial behavior, social skills, self-esteem, emotional introspection, problem-solving, and future/life purpose), and 14 items measure protective contextual factors (adult support at home, adult support at school, and peer support).

##### 2.4.1.1 Additional measures for validation

To provide evidence of relations to external variables, two complementary instruments were administered alongside the RES-PRIM. These measures were selected because they assess related constructs—stress and emotional understanding—that are theoretically linked to resilience, thereby allowing examination of the external validity of the new scale.

#### 2.4.2 School stress subscale of the children’s daily stress inventory

This scale in Spanish consists of 7 dichotomous items that refer to stressors related to an excess of extracurricular tasks, problems interacting with teachers, low school grades, difficulties relating to classmates, and perceived concentration difficulties (56). It is aimed at children aged 6–12 years. The scale has shown adequate reliability (Cronbach’s alpha = .81). Higher scores indicate greater perceived academic stress.

#### 2.4.3 Test of emotional comprehension

The TEC is a formal and standardized measure of emotional comprehension for children aged 3 to 11 years (57). It evaluates the understanding of the nature, causes, and regulation of emotions (e.g., emotions based on external causes or other mental states such as desires or beliefs). The test consists of 23 illustrated stories, where after a brief story, the participant is asked to choose the correct facial expression (emotion) for the main character from four given options. The emotions presented in the 23 items are: happy, sad, angry, scared, and/or well-being. The Spanish version of the test is currently in the validation phase. Thus, to conduct the present study, one of the authors of the TEC provided the authors with the Spanish version. The test has shown adequate test–retest reliability after 3 months [r(18) = .84], and adequate test–retest correlations after 13 months [r(40) = .64 and r(32) = .54]. Cronbach’s alpha showed values between.61 and.97. Higher scores reflect greater emotional comprehension.

### 2.5 Data analysis

Data analyses were conducted following a sequential process to ensure clarity and alignment with the study objectives. (a) Preliminary screening of the initial 40-item pool was carried out using descriptive statistics, corrected item–total correlations, and standardized factor loadings, which guided the elimination of two items with low correlations and weak loadings. In addition, an initial exploratory check of standardized factor loadings from the preliminary measurement model indicated that these two items had weak loadings (<.30), confirming their limited contribution. All subsequent analyses were therefore based on the final 38-item version. (b) Descriptive statistics (means, standard deviations, and frequency distributions) and normality checks (Z= .665; p = .150) were then calculated for the overall scale and each dimension. (c) Evidence of validity regarding the internal structure was examined through Confirmatory Factor Analysis (CFA). Given the Likert-type response format, polychoric correlation matrices and robust maximum likelihood estimators were employed. Model fit quality was assessed using chi-square (χ²), standardized chi-square (χ²/df), p-value of chi-square, Root Mean Square Error of Approximation (RMSEA), Comparative Fit Index (CFI), Tucker-Lewis Index (TLI), and Standardized Root Mean Square Residual (SRMR), applying established interpretation ranges (58, 59): χχ²/df ≤ 3; RMSEA ≤.05 excellent, ≤.08 acceptable; SRMR ≤.08 acceptable; CFI/TLI ≥.90 good, ≥.95 excellent. (d) Internal consistency was analyzed using Cronbach’s alpha and McDonald’s omega. The interpretation of factor loadings was conducted separately within the CFA, as part of the evaluation of the measurement model. Standardized loadings ≥.30 were considered acceptable and ≥.50 strong (59, 60). (e) Evidence of relations to external variables was obtained through Pearson correlations between RES-PRIM scores and the IECI and TEC, as theoretically related measures. (f) Finally, exploratory group comparisons (sex, age, and diagnostic status: TD vs. NDD) were performed using Student’s t-tests and Analyses of Variance (ANOVAs), with effect sizes (Cohen’s d, η²) reported. All analyses were conducted with IBM SPSS Statistics version 29 (IBM Corporation, New York, USA) and IBM Amos version 29, in accordance with the *Standards for Educational and Psychological Testing* (AERA, APA, NCME, 2014) and contemporary approaches that conceptualize validity as an ongoing process of argumentation and evidence gathering (61, 62).

## 3 Results

### 3.1 Psychometric evaluation

#### 3.1.1 Evidence regarding internal structure

To provide evidence of validity regarding the internal structure of RES-PRIM, three factorial models were tested to determine the optimal structure of RES-PRIM. All CFA models were tested using the final 38-item version of the scale, after two items were removed in preliminary analyses due to corrected item–total correlations below.30 and weak standardized loadings (<.30) in the preliminary measurement model. The first evaluated model included two first-order factors (individual resilient factors and contextual resilient factors) and one second-order factor (Resilience). However, Model 1 did not show an adequate fit (**Table 2**), largely due to the very high correlations observed between the individual and contextual first-order factors, which indicated limited independence and low discriminant validity.

**Table 2:** Values of the indices used to assess model fit.

Model	χ^2^	df	*p*	χ^2^/df	CFI	TLI	RMSEA	SRMR
Model 1	78.97	42	.012	2.14	.793	.759	.087	.0934
Model 2	62.85	39	.009	1.88	.901	.892	.052	.0625
Model 3	36.49	17	.000	1.61	.934	.922	.038	.0453

χ², chi-square; df, degrees of freedom; p, overall model significance; χ²/df, standardized chi-square; CFI, Comparative Fit Index; TLI, Tucker-Lewis Index; RMSEA, Root Mean Square Error of Approximation; SRMR, Standardized Root Mean Square Residual.

Model 2 demonstrated an improved fit, but redundancy among dimensions was still evident: several factors showed very high inter-factor correlations and some items presented lower standardized loadings, suggesting overlapping content between certain dimensions.

Finally, a third model was evaluated, consisting of nine first-order factors and two interrelated second-order factors (individual and contextual resilience factors). This model showed the best fit indices (χ²/df = 1.61, RMSEA= .038, SRMR= .0453, CFI= .934, TLI= .922), all within the acceptable to excellent interpretation ranges (58, 59). The covariances between factors ranged from.32 to.89, indicating moderate to high associations between dimensions. The structure of the models is illustrated in **Figure 3**, and the fit values are presented in **Table 2**.

**Figure 4:**
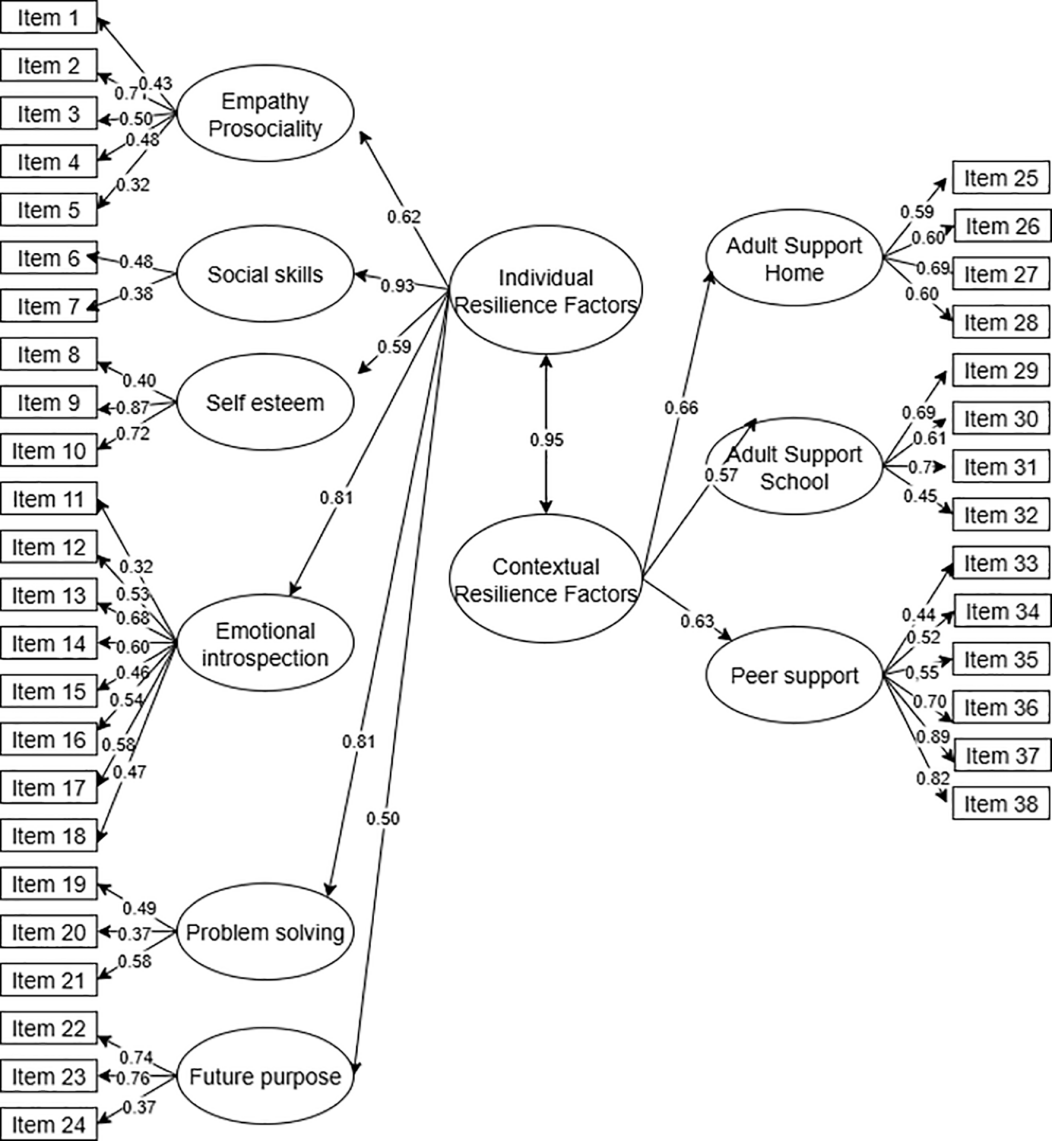
Diagram of Model 3: two interrelated second-order factors and nine first-order factors of the RES-PRIM (final 38-item version). Items are labeled as Item 1–Item 38 to match tables. Standardized factor loadings for all items and for second-order to first-order factors are shown (all significant at p <.001). Item-level error terms are omitted for clarity.

As part of the CFA results, all standardized factor loadings were also examined to evaluate the adequacy of the measurement model. RES-PRIM assesses both individual resilient factors (Empathy/prosociality, Social skills, Self-esteem, Emotional introspection, Problem-solving, and Future purpose) and contextual resilient factors (Adult support at home, Adult support at school, and Peer support). Standardized factor loadings for all items are reported in **Figure 4**, and all met or exceeded the recommended minimum of.30 (59, 60), with most falling in the moderate to high range, thus supporting the adequacy of the items as indicators of their respective factors. Although some dimensions presented comparatively lower loadings (e.g., Future Purpose and Self-esteem), these values are still above the conventional acceptability threshold and were theoretically justified as core dimensions of resilience in childhood. Therefore, they were retained in the final model to preserve the conceptual integrity of the construct.

#### 3.1.2 Evidence of relations to external variables

Evidence of test–criterion relationships was obtained by calculating Pearson correlations between the RES-PRIM scale and other theoretically related variables, such as academic stress and emotional understanding. **Table 3** presents the obtained correlations, highlighting that resilience negatively correlates with academic stress and positively with emotional understanding.

**Table 3:** Pearson correlations (r) between the total RES-PRIM scale and its dimensions, with academic stress and emotional understanding.

RES-PRIM (dimensions/factors)	Academic stress		Emotional understanding	
	*P*	*r*	*P*	*r*
Empathy/Prosociality	-.200**	<.001	.163**	<.001
Social Skills	-.092*	.047	.015*	.743
Self-esteem	-.292**	<.001	.005	.915
Emotional Introspection	-.304**	<.001	.149*	.015
Problem-solving	-.206**	<.001	.013*	.783
Future Purpose	-.100*	.031	.069	.137
Adult Support at Home	-.100*	.032	.035*	.446
Adult Support at School	-.225**	<.001	.177**	<.001
Peer Support	-.326**	<.001	.083*	.075
Individual Resilient Factors	-.327**	<.001	.078*	.094
Contextual Resilient Factors	-.327**	<.001	.130**	.005
RES-PRIM	-.361**	<.001	.110*	.017

* = p <.05.** = p < .001.

Specifically, individual and contextual resilience factors showed significant relationships with academic stress, suggesting that children with higher levels of resilience experience less stress in the school environment. Regarding emotional understanding, a positive correlation was observed with both individual and contextual dimensions; however, no significant relationship was found with self-esteem and future purpose.

#### 3.1.3 Reliability

The reliability of the scale was analyzed using Cronbach’s alpha and McDonald’s omega coefficients. The values obtained for RES-PRIM and each of its dimensions are presented in **Table 4**. As observed, all individual and contextual factors, as well as the overall scale, exhibit satisfactory internal consistency, with values exceeding.70 in most cases.

**Table 4:** Cronbach’s alpha and McDonald’s omega for the internal consistency of each dimension.

RES-PRIM (dimensions/factors)	Cronbach’s alpha	McDonald’s omega
Individual Resilient Factors	.823	.812
*Empathy/Prosociality*	.712	.714
*Social Skills*	.701	.701
*Self-esteem*	.734	.745
*Emotional Introspection*	.826	.811
*Problem-solving*	.758	.784
*Future Purpose*	.722	.738
Contextual Resilient Factors	.824	.813
*Adult Support at Home*	.705	.709
*Adult Support at School*	.703	.710
*Peer Support*	.824	.831
RES-PRIM	.869	.863

#### 3.1.4 Item and dimension analysis

A detailed analysis of each item and dimension was conducted to examine their performance within the sample. Overall, the mean score of the global scale was high (*M*=91.40, *SD*=11.81), indicating that children demonstrate elevated levels of resilience. This trend was observed in both individual and contextual factors, although contextual factors obtained higher scores compared to individual factors.

The disaggregated data for each dimension, along with mean values and standard deviations, are presented in **Table 5**. These results help identify which aspects of resilience are more prevalent in the sample and which could be the focus of specific interventions in future studies.

**Table 5:** Descriptive analysis of items and dimensions of RES-PRIM in children with TD and with NDD.

RES-PRIM (items)		*TD*		*NDD*	
	Range	*M*	*SD*	*M*	*SD*
Individual Resilient Factors	72	55.72	7.51	47.8	7.06
*Empathy/Prosociality*	*0-15*	11.03	2.01	9.4	1.9
1. Si alguien que conozco está triste, yo también me pongo triste./c*1. If someone I know is sad, I also feel sad.*	0-3	1.21	.821	1.06	.68
2. Si a alguien que conozco y me cae bien le pasa algo bueno, me pongo contento./*2. If something good happens to someone I know and like, I feel happy.*	0-3	2.35	.679	2.28	.67
3. Si alguien que conozco tiene un problema intento ayudarle./*3. If someone I know has a problem, I try to help him/her.*	0-3	2.62	.520	2.23	.68
4. Me gusta ayudar a la gente que conozco./*4. I like helping people I know.*	0-3	2.64	.531	2.06	.58
5. Cuando alguien me cuenta algo, escucho con atención lo me dice./*5. When someone tells me something, I listen carefully to what they* are saying.	0-3	2.29	.678	1.77	.42
*Social Skills*	*0-6*	4.75	1.12	4.1	1.2
6. Sé cómo hacer amigos/as nuevos./*6. I know how to make new friends*.	0-3	2.32	.742	1.89	.79
7. Me gusta trabajar con mis compañeros de clase./*7. I enjoy working with my classmates.*	0-3	2.45	.700	2.23	.72
*Self-esteem*	*0-9*	7.26	1.33	6.2	1.1
8. Soy capaz de hacer las cosas bien sin que me tengan que ayudar./*8. I am capable of doing things well without needing help.*	0-3	2.03	.641	1.75	.56
9. Me gusta como soy./*9. I like myself.*	0-3	2.62	.649	2.25	.66
10. Me acepto como soy./*10. I accept myself.*	0-3	2.62	.605	2.28	.70
*Emotional Introspection*	*0-24*	19.06	3.19	16.3	3.3
11. Sé lo que necesito en cada momento para estar bien. *11. I know what I need at any given moment to feel good.*	0-3	2.21	.695	2.17	.74
12. Sé cuándo estoy triste./*12. I know when I am sad.*	0-3	2.61	.592	2.56	.56
13. Sé cuándo estoy contento/*13. I know when I am happy.*	0-3	2.85	.389	2.23	.81
14. Sé cuándo estoy enfadado./*14. I know when I am angry.*	0-3	2.63	.624	2.19	.90
15. Sé cuándo tengo miedo/*15. I know when I feel afraid.*	0-3	2.59	.729	1.36	.88
16. Cuando estoy enfadado, sé que hacer para dejar de estarlo./*16. When I am angry, I know what to do to stop being angry.*	0-3	1.78	1.51	1.67	.92
17. Cuando estoy triste, sé que hacer para dejar de estarlo./*17. When I am sad, I know what to do to stop being sad.*	0-3	1.92	.798	2.33	.47
18. Sé qué cosas me hacen sentir bien./*18. I know what things make me feel good.*	0-3	2.62	.640	2.17	.74
*Problem-solving*	*0-9*	6.67	1.60	5.6	1.4
19. Cuando algo me sale mal, intento hacerlo de otra manera para que me salga bien./*19. When something goes wrong, I try to do it differently to make it work.*	0-3	2.28	.760	1.91	.72
20. Cuando algo me sale mal, sé a quién puedo pedirle ayuda./*20. When something goes wrong, I know who I can ask for help*.	0-3	2.35	.725	1.94	.83
21. Cuando tengo un problema con alguien sé buscar una solución./*21. When I have a problem with someone, I know how to find a solution*.	0-3	2.12	.774	1.81	.81
*Future Purpose*	*0-9*	6.92	1.87	6	1.7
22. Pienso en qué me gustaría estudiar cuando sea más mayor./*22. I think about what I would like to study when I am older.*	0-3	2.24	.885	1.70	.83
23. Pienso en qué cosas me gustaría hacer cuando sea mayor./*23. I think about the things I would like to do when I am older*.	0-3	2.24	.852	1.97	.79
24. Pienso que cuando sea mayor seré feliz./*24. I think that when I am older, I will be happy.*	0-3	2.45	.744	2.33	.73
Contextual Resilient Factors	0-42	35.59	5.46	30.1	4,6
*Adult Support at Home*	*0-12*	11.01	1.54	9.5	1,8
25. Hay algún adulto que me cuida cuando no me encuentro bien./*25. There is an adult who takes care of me when I am not feeling well.*	0-3	2.87	.431	2.48	,73
26. Hay algún adulto con el que puedo hablar y contarle mis problemas./*26. There is an adult I can talk to and tell my problems to.*	0-3	2.63	.599	2.13	,88
27. Hay algún adulto que me dice que puedo hacer las cosas bien./*27. There is an adult who tells me I can do things well.*	0-3	2.76	.542	2.41	,75
28. Tengo algún adulto en casa que me dice lo que está bien y lo que está mal./*28. There is an adult at home who tells me what is right and what is wrong.*	0-3	2.79	.484	2.48	,64
*Adult Support at School*	*0-12*	7.80	1.58	9.1	1.8
29. Hay un profesor/a que me ayuda cuando algo no me sale bien./*29. There is a teacher who helps me when something doesn’t go well.*	0-3	2.57	.626	2.22	.74
30. Hay un profesor/a a quien puedo contarle un problema./*30. There is a teacher I can talk to about a problem.*	0-3	2.31	.775	2.08	.74
31. Hay un profesor que me dice que puedo hacer bien las cosas del cole./*31. There is a teacher who tells me I can do well at school.*	0-3	2.45	.730	2.34	.67
32. Hay un profesor que me dice lo que está bien y lo que está mal./*32. There is a teacher who tells me what is right and what is wrong.*	0-3	2.62	.595	2.38	.60
*Peer Support*	*0-18*	14.67	3.52	11.6	3.4
33. Hay compañeros/as que juegan conmigo./*33. There are classmates who play with me.*	0-3	2.71	.583	2.23	.72
34. Hay compañeros/as que me ayudan cuando lo necesito./*34. There are classmates who help me when I need it.*	0-3	2.53	.636	1.88	.70
35. Hay compañeros/as con quien hablar de nuestras cosas./*35. There are classmates with whom I can talk about personal things.*	0-3	2.62	.684	1.92	.76
36. Tengo algún amigo/a con el que juego./*36. I have a friend with whom I play.*	0-3	2.46	.858	2.17	1.1
37. Tengo algún amigo/a que me ayuda cuando lo necesito./*37. I have a friend who helps me when I need it.*	0-3	2.19	.951	1.73	.99
38. Tengo algún amigo/a con quien hablar de nuestras cosas./*38. I have a friend with whom I can talk about personal things.*	0-3	2.24	1.01	1.67	.87
RES-PRIM	0-114	91.40	11.81	77.9	10.5

#### 3.1.5 Evidence from inter-group comparisons (TD vs. NDD)

To analyze differences between children with typical development (TD) and children with neurodevelopmental disorders (NDD), Student’s t-tests were conducted. As shown in **Table 6**, children with NDD obtained significantly lower scores in both individual and contextual resilience factors compared to children with TD (p <.001), with large effect sizes in both comparisons.

**Table 6:** Inter-group comparisons between children with TD and children with NDD.

RES-PRIM (factors and total score)	TD	NDD	*p*	*d*
	*M (SD)*	*M (SD)*		
Individual Resilient Factors	55.4 (6.4)	47.8 (7.1)	<.001	1.2
Contextual Resilient Factors	36.1 (3.8)	30.1 (4.6)	<.001	1.8
RES-PRIM	91.5 (9.1)	77.9 (10.5)	<.001	1.5

### 3.2 Analysis of demographic differences (age and sex)

Gender differences in resilience levels were explored using Student’s t-tests. As shown in **Table 7**, no significant differences were found between boys and girls in the total scale or in individual and contextual resilience factors, suggesting that resilience manifests similarly across both genders within the analyzed sample.

**Table 7:** Sex differences.

RES-PRIM (factors and total score)	Boys	Girls	*p*	*d*
	*M (SD)*	*M (SD)*		
Individual Resilient Factors	54.86 (7.37)	57.23 (7.05)	.109	-,328
Contextual Resilient Factors	35.01 (5.27)	36.52 (5.31)	.744	-,288
RES-PRIM	89.86 (11.39)	93.76 (11.22)	.293	-,344

Finally, an ANOVA was conducted to examine differences in resilience based on age. **Table 8** shows that significant differences were found in the total scale and in both individual and contextual resilience factors across different educational levels (p <.001).

**Table 8:** Age differences presented by course.

RES-PRIM (factors and total score)	Course *M (SD)*						F	*p*	*η²*
	1	2	3	4	5	6			
Individual Resilient Factors	59.26 (6.68)	58.38 (8.66)	56.55 (7.13)	54.34 (7.35)	53.41 (6.84)	53.71 (5.08)	9.70	<.001	.096
Contextual Resilient Factors	36.78 (4.96)	37.12 (8.66)	35.02 (4.45)	35.02 (5.99)	34.08 (5.92)	37.12 (4.25)	3.78	.002	.046
RES-PRIM	96.04 (10.81)	95.51 (13.21)	89.12 (9.98)	90.36 (12.64)	87.50 (11.14)	95.51 (8.10)	7.57	.001	.076

Overall, first- and second-grade children exhibited the highest levels of resilience, whereas fifth-grade students showed lower scores compared to other groups.

These findings suggest that resilience may vary throughout childhood development, possibly due to changes in environmental perception, autonomy, and emotional regulation.

## 4 Discussion

The objectives of this study were to design and provide evidence of validity and reliability for the RES-PRIM as a multidimensional measure of resilience in primary-school children, including those with and without neurodevelopmental disorders. Findings confirmed evidence of validity, reliability, and relations with external variables, supporting the adequacy of the instrument, in line with current perspectives that emphasize validity and reliability as ongoing processes rather than absolute outcomes (61, 62). By incorporating both individual and contextual factors, RES-PRIM offers a comprehensive framework for assessing resilience in inclusive educational settings.

From a theoretical perspective, resilience can be understood as a dynamic process of positive adaptation in the context of adversity, shaped by the interplay between individual assets and environmental supports (17, 62). This systemic and ecological conceptualization is particularly relevant for neurodivergent populations, as it emphasizes that resilience is not only rooted in personal traits but also in the accessibility of social, family, and educational resources.

Rather than reiterating the statistical indices already presented in the Results, the Discussion emphasizes the theoretical and practical implications of these findings. The good fit of the models provides strong evidence regarding the internal structure of the RES-PRIM, supporting its adequacy as a multidimensional measure of resilience. This suggests that resilience can be meaningfully represented through both individual and contextual factors, reinforcing the idea that children’s coping capacities are shaped not only by personal skills but also by social supports. These results are consistent with previous studies highlighting the ecological and systemic nature of resilience (12, 17). Taken together, these results reinforce the validity of the RES-PRIM as a robust instrument for assessing resilience in primary school children. Importantly, the findings indicate that RES-PRIM can serve as a reliable tool for identifying protective profiles that integrate both personal and environmental dimensions, which is particularly relevant for informing inclusive educational practices and designing targeted interventions.

A significant negative association was found between resilience and school-related stress, both in the overall resilience scale and in individual and contextual resilience factors. This finding underscores the importance of resilience as a coping mechanism for academic stress, consistent with previous studies (38, 39). Lower stress perceptions translate into better daily functioning, suggesting that by strengthening RES-PRIM factors practitioners may not only buffer adversity but also elevate children’s subjective QoL in inclusive settings. Additionally, variables such as self-esteem, self-concept, social skills, problem-solving, and emotional regulation were also significantly associated with school stress, aligning with the existing literature (63). Notably, a positive correlation was found between empathy/prosociality and school stress, an unexpected result in the school context. One possible interpretation is that highly empathic children, while socially attuned, may also be more prone to experiencing vicarious stress or emotional contagion when perceiving distress in others. Previous studies have suggested that empathy, although generally protective, can increase susceptibility to stress and emotional burden in highly sensitive individuals (64, 65). This interpretation highlights the need for further research, as interventions should aim to foster prosocial empathy while simultaneously equipping children with strategies to regulate the potential emotional costs of heightened empathic responsiveness.

Regarding contextual resilience factors, perceived teacher, parental, and peer support was significantly associated with lower perceptions of school stress, emphasizing the importance of social support in both family and educational settings to foster child resilience, in line with previous research (63, 66). Such multilayered support mechanisms mirror the “environmental facilitators” as essential pathways to inclusion and well-being.

Furthermore, a positive correlation was found between resilience and emotional understanding. This indicates that higher emotional regulation and comprehension are associated with higher levels of resilience in children, as suggested by previous studies (67). When analyzing this relationship by factor type, emotional understanding was significantly related to social interaction dimensions (contextual factors, empathy/prosociality, social skills, and problem-solving), but not to individual skills such as self-esteem or future life purpose.

The data indicate a medium-high level of resilience in the overall group of students, both in the global score and by factors. However, when comparing TD and NDD groups, it was observed that resilience levels were lower in the NDD group, both in individual and contextual resilience factors. This finding aligns with previous studies analyzing resilience in young people with NDD, identifying lower levels, particularly regarding social support, but also in individual variables such as self-perception (9, 15, 22). These disparities reinforce the need for targeted, strengths-based interventions—guided by RES-PRIM results—that can close QoL gaps and promote equitable participation in mainstream classrooms.

On the one hand, lower values in individual factors obtained by children with NDD may be due to the inherent difficulties of neurodevelopmental disorders. However, with adequate support, these children can develop resilience skills that enhance their coping and adaptability, although this process may require more time and effort (10). On the other hand, lower values in contextual factors suggest that children with NDD may require a more structured and predictable environment to strengthen their resilience skills. A stable family environment, adequate school support, and access to therapeutic interventions are key aspects for their development (9, 22). Embedding such supports within inclusive policy frameworks will be critical for translating assessment insights into sustainable QoL gains, echoing recommendations from recent scoping reviews on resilience in neurodivergent populations (68).

Regarding gender differences, no significant differences were found in resilience levels, which contrasts with some studies suggesting higher resilience levels in girls as they grow older (69). One possible explanation is that gender differences in resilience may emerge more clearly in adolescence, when socialization processes and self-concept become more differentiated (70). In addition, cultural factors may play a role: in the Spanish educational context—where gender equality and co-educational practices are strongly promoted—gender gaps in perceived resilience may be minimized; this is aligned with findings from the Spanish adaptation of the CYRM-32 scale, which revealed no gender differences among children and adolescents (71). Finally, the use of a child-friendly self-report instrument with visual supports, such as the RES-PRIM, may reduce potential gender biases by facilitating comprehension for both boys and girls. This finding therefore highlights the importance of conducting longitudinal studies to examine whether gender-related patterns in resilience become more pronounced at later developmental stages.”

In terms of differences by school grade, a decrease in resilience levels was observed as children progressed through their education. This trend may be related to cognitive, emotional, social, and moral development, as resilience is a dynamic process that evolves with age (72). Longitudinal monitoring with RES-PRIM could help schools anticipate these developmental dips and proactively design tiered supports that sustain children’s well-being through transitional periods (73).

## 5 Limitations and future directions

Although this study represents a significant advancement in assessing resilience in childhood, some limitations must be considered. First, the study employed a non-probabilistic convenience sampling method, and all participating schools came from a single region. This may restrict the generalizability of the findings to other cultural or educational contexts. Future studies should include samples from different countries and larger, more diverse populations to increase representativeness.

Second, although RES-PRIM provided evidence of validity and reliability in children with and without NDD, the subgroup of children with NDD was relatively small, and the number of participants with ASD in particular was limited. In addition, children with ASD and ADHD were considered together in the same group, which prevents drawing condition-specific conclusions. These aspects suggest that the results for the NDD group should be interpreted with caution and considered preliminary. Future research should replicate provision of validity evidence with larger and diagnostically differentiated groups, as well as conduct measurement invariance and differential item functioning analyses to test the robustness of the instrument across populations.

Third, a longitudinal follow-up is recommended to assess the temporal stability of the scale and determine how resilience evolves over time. Future studies could also analyze the effectiveness of interventions based on RES-PRIM, measuring changes in child resilience after the implementation of educational or therapeutic programs designed using the scale’s results.

Finally, the study did not control for other variables that may influence resilience development, such as socioeconomic status, parental mental health, or previous exposure to adverse experiences. Future research should incorporate a more comprehensive analysis of contextual and environmental factors to better understand their impact on resilience. Moreover, participatory research methods involving children, families, and educators could ensure that subsequent adaptations of RES-PRIM remain sensitive to the lived experiences and priorities of neurodivergent learners.

Despite these limitations, RES-PRIM offers an innovative approach to assessing child resilience, particularly in young children and those with NDD, making it a valuable tool for both research and educational applications.

## 6 Conclusion

RES-PRIM is a child-friendly and easy-to-administer instrument, as it can be applied both individually with younger children and in group classroom sessions with older students, requiring only 20–40 minutes depending on grade level. Built on solid theoretical foundations and demonstrating adequate psychometric evidence, it offers a valuable tool for assessing resilience capacities in children with and without NDD. However, the findings for the NDD group should be interpreted with caution given the relatively small sample size and the grouping of ASD and ADHD participants. Further research with larger and diagnostically differentiated samples is therefore required to strengthen these conclusions.

Crucially, the present findings position RES-PRIM as a bridge between assessment and action: the scale not only captures resilience profiles but also generates data that can guide multi-tiered supports designed to enhance children’s QoL and inclusion in mainstream education. The availability of scales like RES-PRIM is crucial for understanding how resilience skills develop throughout primary education and for detecting at an early stage which children may need additional support in developing resilience, particularly those at risk of experiencing adverse situations, such as peer rejection or increased awareness of their own difficulties due to NDD. By spotlighting both strengths and needs, RES-PRIM empowers educators, clinicians, and policymakers to craft evidence-based interventions that foster pathways to well-being and full participation for all children, irrespective of neurodevelopmental profile. In this regard, social support is essential during this stage of development. Furthermore, this instrument will enable the design of personalized interventions, addressing both individual and contextual aspects of resilience, and facilitating the creation of group programs with active involvement of teachers and families.

## Data Availability

The datasets presented in this study are publicly available. This data can be found here: http://hdl.handle.net/10234/742894 (Database of individual and contextual resilience factors in primary school children with and without neurodevelopmental disorders).
