# Correction: Design and psychometric evaluation of RES-PRIM: a resilience scale for primary education students with and without neurodevelopmental disorders

**DOI:** 10.3389/fpsyt.2025.1722457

**Published:** 2025-10-22

**Authors:** Raquel Flores-Buils, Clara Andrés-Roqueta, Rosa Mateu-Pérez

**Affiliations:** ^1^ Department of Developmental, Educational Social and Methodological Psychology, Universitat Jaume I, Castellón de la Plana, Spain; ^2^ Department of Pedagogy and Didactics of Social Sciences, Language and Literature, Universitat Jaume I, Castellón de la Plana, Spain

**Keywords:** resilience, children, primary education, typical development, neurodevelopmental disorders, individual and contextual protective factors, quality of life, psychometric evaluation

There was a mistake in **Figure 3** as published. The right panel was missing the English translation of the Likert scale. The corrected **Figure 3** appears below.

The figures were in the wrong order in the paper. **Figures 1** and **3** were swapped, though their captions were correct. The order of the figures has now been corrected.

**Figure 1:**
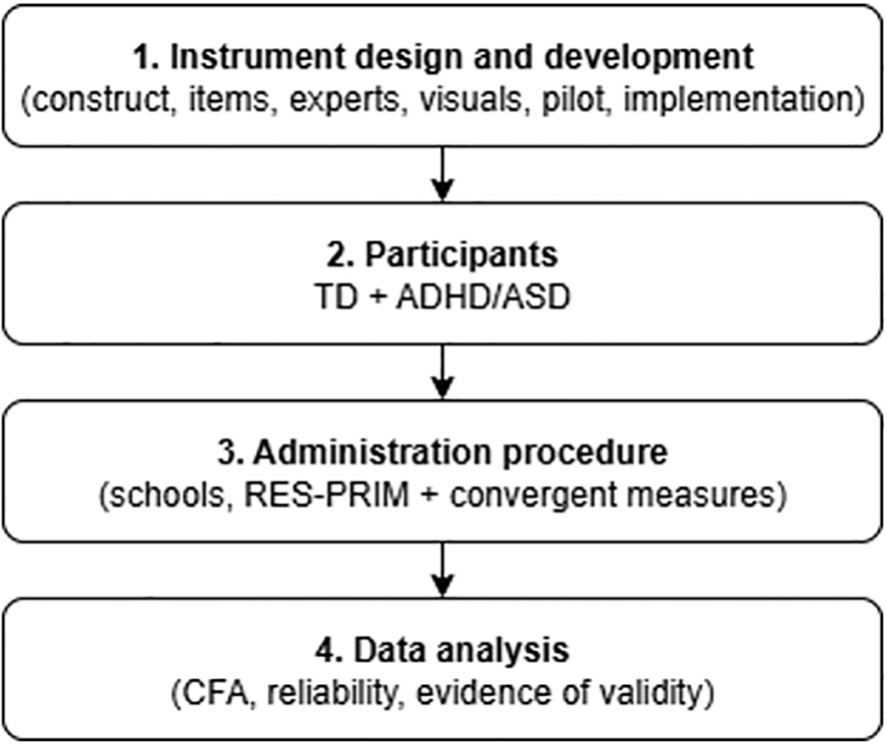
Methodological sequence of the study.

**Figure 3:**
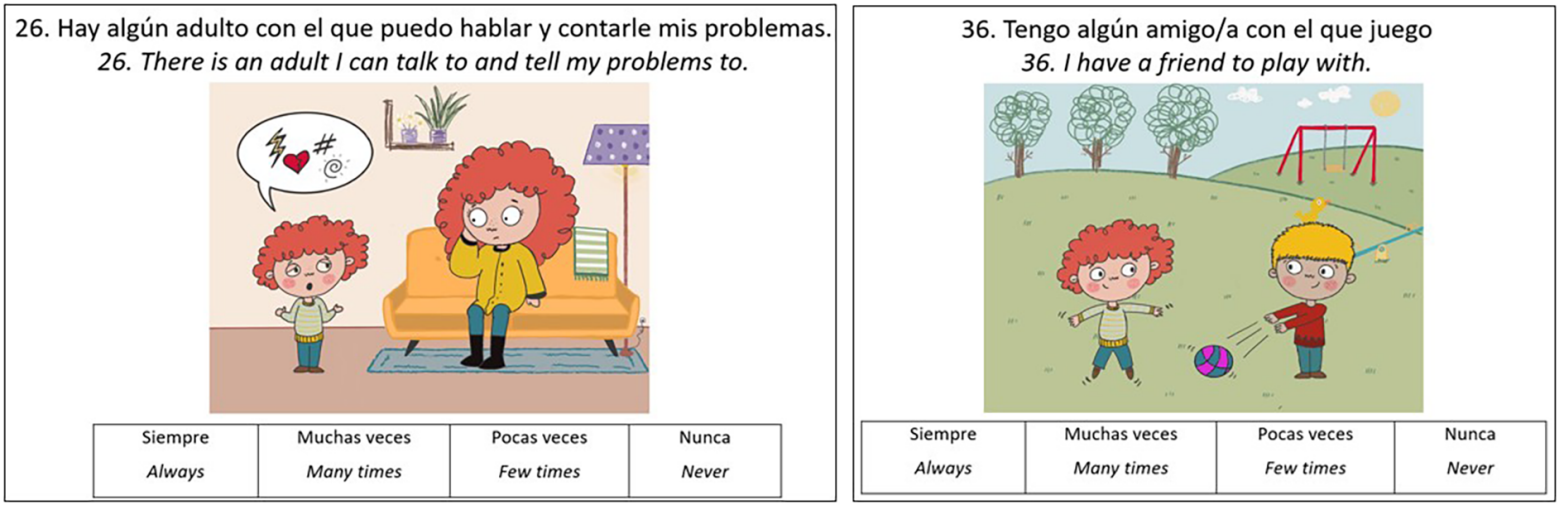
Examples of items on contextual factors.

The original version of this article has been updated.

